# Delayed Dexamethasone Absorption from Gluteal Abscesses: Persistent Iatrogenic Cushing Syndrome

**DOI:** 10.1177/11795514251376453

**Published:** 2025-12-01

**Authors:** Domas Grigoravicius, Karolis Laucius, Rasa Ugenskiene, Vaida Stankute, Zydrune Visockiene

**Affiliations:** 1 Institute of Clinical Medicine, Faculty of Medicine, Vilnius University, Lithuania; 2 Genetic and Molecular Medicine Clinic, The Hospital of Lithuanian University of Health Sciences Kauno Klinikos, Kaunas, Lithuania

**Keywords:** iatrogenic Cushing syndrome, dexamethasone, abscess fluid steroid profile

## Abstract

Iatrogenic Cushing syndrome can occur due to the use of external glucocorticoids. We present a case involving prolonged exposure to glucocorticoids from intramuscular dexamethasone injections, with diagnosis confirmed via abscess fluid analysis. This underscores the importance of considering altered pharmacokinetics when faced with unexplained hypercortisolism. A 46-year-old woman experienced hypertension, weakness, edema, and increasing abdominal striae over 6 months. Her history included undocumented intramuscular injections for back pain a year earlier, which resulted in bilateral gluteal abscesses. Laboratory results showed suppressed morning cortisol and adrenocorticotrophic hormone levels, while imaging ruled out structural abnormalities. Although serum tests were negative, liquid chromatography–mass spectrometry performed on abscess fluid detected dexamethasone (81.1 nmol/L), confirming iatrogenic Cushing syndrome. This case highlights how local tissue changes, such as abscesses, can significantly modify glucocorticoid pharmacokinetics, creating a prolonged reservoir effect and sustained systemic exposure lasting more than a year after injection. Overall, atypical pharmacokinetics are important in cases of unexplained hypercortisolism, especially when local tissue alterations influence drug absorption and clearance. Analyzing collections like abscess fluid can provide vital diagnostic clues in complex suspected cases of iatrogenic Cushing syndrome.

## Plain language summary

A rare cause of long-lasting steroid side effects from an old injection

Cushing syndrome is a condition caused by excessive levels of the hormone cortisol in the body. This can result in symptoms like weight gain, high blood pressure, and an overall decline in well-being. The most common cause of this condition is the use of steroid medications, known as glucocorticoids, for various health issues. This type is called iatrogenic Cushing syndrome.

In this report, we describe a rare and unusual case. A 46-year-old woman developed Cushing syndrome more than a year after receiving several undocumented injections for back pain. These injections, administered into the muscles of her buttocks, later caused abscesses (collections of pus) to form at the injection sites. Over time, she developed symptoms of Cushing syndrome, but standard blood and urine tests could not find any steroids in her system.

Further investigation revealed the answer: advanced testing of fluid from one of her abscesses detected dexamethasone, a strong synthetic steroid. The results showed that the drug had been slowly leaking into her bloodstream from the abscess for many months—far longer than expected. After the abscess was drained, she developed temporary adrenal gland underactivity, which required steroid replacement. Over the following year, her hormone levels returned to normal, and her symptoms improved.

This case shows that steroid medicines can sometimes remain trapped in body tissues, such as abscesses, and be released very slowly over time, causing prolonged side effects. It also highlights the value of testing unusual samples, like abscess fluid, when standard tests are negative but suspicion for steroid exposure remains high.

Understanding this rare situation can help physicians diagnose and treat unexplained cases of Cushing syndrome more effectively.

## Introduction

Iatrogenic Cushing syndrome (CS) is a condition characterized by high cortisol levels caused by the use of external glucocorticoids (GC).^1^ The prevalence of iatrogenic CS outweighs endogenous CS because glucocorticoids are widely used in medicine and are available as over-the-counter medications.^2^ The reliance on these medications makes iatrogenic CS the most frequently encountered cause of Cushing syndrome.^1^

Iatrogenic CS can develop while a patient is actively taking glucocorticoids or may appear after the treatment has stopped.^3,4^ The timing of the onset of symptoms depends on the specific drug elimination speed and method of administration, whether taken orally, topically, intravenously, or as an injection.^4,5^ Also, the elimination speed of GCs varies from patient to patient.^4^ Nevertheless, diagnosing iatrogenic CS is challenging, even considering the differences in GC pharmacokinetics and individual variability. Sometimes, it requires reviewing the possibilities of obstructed GC absorption or elimination.^1,3,6^

We present a case highlighting the importance of considering atypical pharmacokinetics in iatrogenic Cushing syndrome, especially when local tissue changes may impact glucocorticoid absorption and elimination. Furthermore, it showcases the diagnostic value of LC-MS/MS steroid profiling for analyzing fluid collections to understand the cause of hypercortisolism.

## Case Presentation

A 46-year-old premenopausal woman presented to the Endocrinology department with hypertension, generalized weakness, ankle and tibial swelling, proximal myopathy, and pink-red striae on the abdomen and breasts. Her symptoms had progressed gradually over 6 months.

Her history was notable for severe lower back pain beginning a year prior, treated with undocumented intramuscular injections administered by nursing home coworkers for pain management. The patient could not recall the details of the injections, and no medical records were available. Bilateral abscesses developed at the injection sites, necessitating abscess drainage. Before the operation, the patient was sent to an endocrinologist for evaluation of suspected secondary hypertension. She denied using any other form of steroids, including topical steroids.

**Figure 1.**
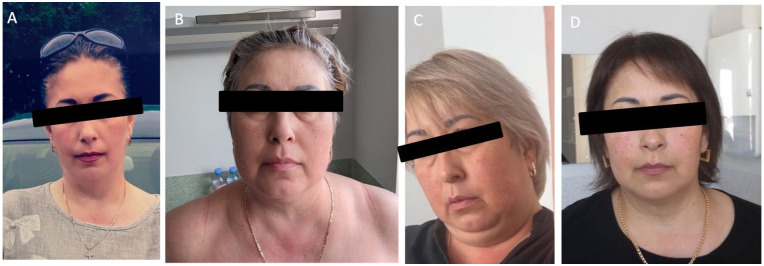
The patient facial features before the presentation (A), on the first inpatient stay (B), on the last inpatient stay (C), and the improvement of the facies lunata after the treatment (D).

**Figure 2.**
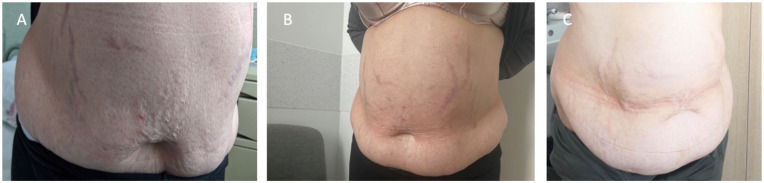
The patient striae progression from the first visit (A) to the last visit (B), and the improvement of striae and adiposity after the treatment (C).

On examination, the patient exhibited central obesity (BMI 36.1 kg/m^2^), a blood pressure of 200/120 mmHg, and a pulse of 96 bpm. Bluish striae were observed bilaterally on the abdomen, breasts, and thighs, with multiple ecchymotic patches on the limbs (Figures 1 and 2). Thyroid palpation and heart and lung auscultation were unremarkable. She reported regular menstrual cycles and had 2 healthy children. There was no family history of diabetes or obesity. The previous medical report documented osteopenia. Laboratory tests revealed suppressed cortisol and ACTH levels (Table 1, column “Initial values”), prompting her admission to the Endocrinology Department.

**Table 1.** Patients’ Initial and Follow-Up Laboratory Values.

Laboratory measure	Initial values	Endocrinology department	6 mo after	8 mo after	12 mo after	Normal value
ACTH	<5 pg/mL (<5 ng/L)	<5 pg/mL (<5 ng/L)	<5 pg/mL (<5 ng/L)	<5 pg/mL (<5 ng/L)	**47 pg/mL (47 ng/L)**	<46 pg/mL (<46 ng/L)
Morning cortisol	<LOD **µg/dL (0 nmol/L)**	<LOD **µg/dL (0 nmol/L)**	**0.11 µg/dL (3 nmol/L)**	**2.18 µg/dL (60 nmol/L)**	6.78 µg/dL (187 nmol/L)	3.67-19.44 µg/dL (101-536 nmol/L)
DHEA-S	**2.21 µg/dL (0.06 µmol/L)**	—	—	—	—	58.0-297.6 µg/dL (1.5-7.7 µmol/L)
Urine cortisol	<LOD **µg/24 h (0.0 nmol/24 h)**	—	<LOD **µg/24 h (0.0 nmol/24 h)**	—	—	4.3-176.1 µg/24 h (11.8-485.6 nmol/24 h)
Potassium	5.1 mEq/L (5.1 mmol/ L)	4.6 mEq/L (4.6 mmol/L)	4.3 mEq/L (4.3 mmol/L)	5.2 mEq/L (5.2 mmol/L)	—	3.8-5.3 mEq/L (3.8-5.3 mmol/L)
Sodium	139 mEq/L (139 mmol/l)	140 mEq/L (140 mmol/ L)	141 mEq/L (141 mmol/L)	138 mEq/L (138 mmol/L)	—	134-145 mEq/L (134-145 mmol/L)

Abbreviations: ACTH, adrenocorticotropic hormone; DHEA-S, dehydroepiandrosterone sulfate; LOD, limit of detection.

Abnormal values are shown in bold font. Values in parenthesis are International System of Units (SI).

**Figure 3.**
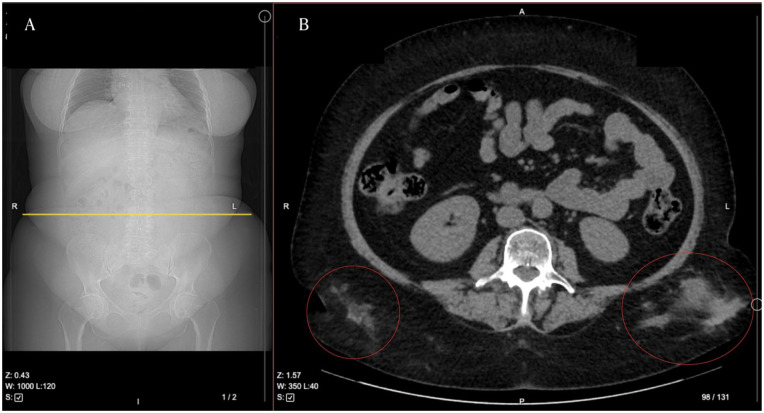
A section of whole-body CT. Yellow line (A) indicates the placement of the axial CT view depicted in (B). Red circles mark an abscesses in the gluteal region. R—right, L—left, A—anterior, P—posterior.

A Synacthen 250 µg stimulation test confirmed axis suppression (cortisol levels: 24 nmol/L at 30 minutes; 31 nmol/L at 60 minutes). Imaging, including pituitary MRI and SPECT, excluded structural pituitary or adrenal abnormalities. Whole-body CT revealed pulmonary artery thromboembolism and residual gluteal abscesses (Figure 3). Due to the embolism, she was transferred to the Intensive Care Unit, where she was also diagnosed with COVID-19, though glucocorticoids were avoided during treatment.

Despite outpatient follow-up, her symptoms worsened, including increased striae, weight gain, weakness, and depression. Persistently suppressed ACTH and cortisol levels led to consideration of factitious Cushing syndrome, cyclic Cushing syndrome, or glucocorticoid receptor mutations. Genetic testing revealed no abnormalities in the NR3C1 gene.

Recurrent febrile gluteal abscesses prompted further investigation. Before the abscess drainage, we obtained abscess fluid, urine and serum samples. Advanced steroid profiling via liquid chromatography-tandem mass spectrometry (LC-MS/MS) detected 874.4 nmol/L (31.7 ng/mL) of dexamethasone in the abscess fluid. However, serum and urine samples were negative. The patient further denied any prior knowledge of the administration of dexamethasone.

After the abscess drainage, the patient displayed symptoms consistent with adrenal insufficiency: low blood pressure of 90/50 mmHg, drowsiness, and intolerance to physical activity. According to institutional protocol, we initiated an infusion of Hydrocortisone at 100 mg per 24 hours.

The patient was discharged on a tapering regimen of prednisolone, starting at 7.5 mg daily. Over the following months, her symptoms improved, with reported increases in energy, weight loss, and normalization of blood pressure. Twelve months after the initial presentation, cortisol after 60 minutes of 250 µg synacthen infusion was 514 nmol/L (Table 1, column “12 months after”), and prednisolone was discontinued. She remains in remission, and follow-up care has been concluded.

## Discussion

In our case, the patient did not use any exogenous steroids except for dexamethasone intramuscular injections that were administered about a year before the clinical presentation. A similar case series was reported by Hughes et al in 1986.^6^ The authors attributed the development of Cushing’s syndrome to the pharmacokinetic characteristics of dexamethasone acetate, which was used in both reviewed cases.^6^ In Lithuania, the only available form of dexamethasone is dexamethasone sodium phosphate, according to the data from Lithuania’s National Drug Control Agency.^7^ The peak concentration of intramuscular dexamethasone phosphate injection is reached in 1 hour after the injection, and the action can last from 17 to 28 days.^7^

To some extent, the timing of the patient’s symptoms could be justified by individual variations in the clearance of dexamethasone, which could vary twofold from person to person.^6^ Although it could explain the initiation of the first symptoms 2 months after the last dexamethasone administration, the condition worsening for the following 2 years for this patient was probably caused by some restriction of dexamethasone elimination.^1^

Another point in this case is the unknown dosage and frequency of dexamethasone administration. However, considering the bilateral gluteal abscesses, injections could have been administered repeatedly.

Given the lack of direct evidence in the literature, we propose that 3 factors may have contributed to the patient’s prolonged exposure following abscess formation: repeated injections leading to tissue saturation, sequestration of dexamethasone within the abscess cavity, and impaired clearance from inflamed tissue. This interpretation is based on extrapolation from similar case reports and data on restricted absorption sites, such as joints.^6,8^ Notably, this aligns with Hughes et al’ observations regarding depot effects of intramuscular steroids; however, our case demonstrates a duration of retention approximately 12 times longer than previously reported.^6^

To our knowledge, the literature does not describe any case of dexamethasone depot in the abscess at the administration site. As a glucocorticoid, dexamethasone should have slowed or even prevented the formation of the abscesses; however, animal model studies have shown that glucocorticoids have a minimal effect on abscess development.^9^

In terms of diagnostics, the sole feasible method to distinguish the iatrogenic cause of Cushing is to identify a synthetic glucocorticoid in serum or urine, which is most effectively achieved using LC-MS/MS.^10-12^ Of course, LC-MS/MS will be diagnostic only if it finds a synthetic glucocorticoid. If not, it is impossible to completely rule out that the patient has been taking medication but was not taking it at the time of analysis by LC-MS/MS.^10^ In our case, dexamethasone was not discovered in the urine or serum by LC-MS/MS. However, it was found in an unusual sample – abscess fluid, thus confirming the dexamethasone depo and reduced absorption theory. Following the abscess drainage, we observed adrenal insufficiency, which further highlights the role of dexamethasone depot in the diagnosis.

## Conclusions

This case underscores the clinical significance of altered pharmacokinetics in iatrogenic Cushing syndrome, particularly when local tissue changes, such as abscess formation, affect drug absorption and clearance. The use of LC-MS/MS for direct analysis of fluid collections proved essential in establishing the diagnosis, especially in cases where standard serum and urine testing may be inconclusive. Our experience highlights the importance of considering localized steroid depots and pursuing comprehensive diagnostic evaluation in patients with refractory or unexplained Cushing syndrome and a history of injectable glucocorticoid therapy.
